# Amino acids disrupt calcium-dependent adhesion of stratum corneum

**DOI:** 10.1371/journal.pone.0215244

**Published:** 2019-04-16

**Authors:** Jin-Hyun Kim, Byungjun Ahn, Seon-Guk Choi, Sohyun In, A. Reum Goh, Sun-Gyoo Park, Cheon-Koo Lee, Nae-Gyu Kang

**Affiliations:** R&D Center, LG Household and Health Care, Ltd., Seoul, Korea; San Gallicano Dermatologic Institute, ITALY

## Abstract

In the stratum corneum, the intercellular junction made up of cadherin proteins provides the structural integrity of the framework. Ca^2+^ ions are known to play a key role in maintaining this junction. In this study, we hypothesized that Ca^2+^ chelation in stratum corneum will weaken the bond of the tissue and consequently promote exfoliation. Amino acids, ubiquitously existing as metabolites and building blocks of the body, have the molecular property to chelate Ca^2+^ ions. In the current study, we verified the Ca^2+^ chelating property of amino acids and demonstrated that amino acids can interfere with the interaction of cadherins, separate stratum corneum into pieces, and thereby stimulate the exfoliation process of skin. These results validate the importance of Ca^2+^ ion in the skin exfoliation process. Importantly, our findings indicate that amino acids may be efficiently used for improving skin conditions.

## Introduction

Stratum corneum is the outermost barrier of the skin that maintains the internal physiological environment and prevents damage from the outside. It consists of corneocytes formed by the differentiation of epidermal keratinocyte cells and intercellular lipid matrix filling between the cornified cells. Corneocytes are interconnected by corneodesmosomes, which are adhesive junctions imparting structural integrity to stratum corneum. The intercellular attachments of corneodesmosomes consist of two transmembrane proteins belonging to the cadherin superfamily, mainly desmoglein1 (Dsg1) and desmocollin1 (Dsc1). Classical cadherins have an overall modular structure of four to five extracellular cadherin (EC) domains and are dimerized by swapping N-terminal β-strands in EC1 domains from opposite cells [1]. Dsg1 and Dsc1 have been reported to bind each other through the ‘strand-swap’ mechanism similar to classical cadherins, except that the binding occurs through heterophilic interaction by opposite charge in the binding interface of Dsg1 and Dsc1 [2]. In the exfoliation process, Dsg1 and Dsc1 are detached and corneocytes are shed into the environment [3].

Ca^2+^ ions are essential for the molecular interaction of cadherin, which is named for ‘calcium-dependent adhesion’. The cadherin has several conserved Ca^2+^-binding pockets composed of glutamate and aspartate in the flexible linker regions between each EC domains. When Ca^2+^ binds to the pockets, the cadherin is structurally modified and stabilized in elongated rod-like form [4,5]. This conformational change narrows the physical distance between the cadherins that extend from the opposite side. Ca^2+^ binds the interface of EC1 and EC2, resulting in strand-swap dimerization, wherein tryptophan residue at position 2 in EC1 docks into a complementary alanine-rich cavity in EC1 of the partner cadherin [1]. Several studies have shown that cadherins reversibly bind and separate depending on the concentration of Ca^2+^ ions [2,6]. Ca^2+^ has also been reported to inhibit the degradation of cadherin by proteases such as Kallikrein-related peptidase (KLK), thereby maintaining the adhesive junction [5,7]. This Ca^2+^ dependence of cadherin association means that the structural integrity of stratum corneum is regulated by Ca^2+^ ions. In normal skin, the highest concentration of Ca^2+^ is maintained in the granular layer below the stratum corneum and the concentration progressively decreases toward the skin surface, leading to natural exfoliation of stratum corneum [8].

Considering the role of Ca^2+^ ions in cadherin structure and function, Ca^2+^ chelators can accelerate the exfoliation process of stratum corneum. Indeed, several studies have shown that EGTA separates the cadherin dimers and takes apart cell aggregates [2,6,9]. As representative molecules, hydroxy acids are also reported to have Ca^2+^ chelating activity as one of the action mechanisms and are widely used to treat calluses, keratoses, acne, psoriasis, and skin changes associated with ageing [10,11]. Molecules with chemical structures containing two or more of the functional groups such as carboxylic acid, hydroxyl group, sulfhydryl group, and amino group, can coordinate with divalent metal ions [12]. In this regard, amino acids are expected to chelate Ca^2+^ ions and stimulate exfoliation of stratum corneum. In this study, we demonstrate that amino acids are able to disrupt Ca^2+^-mediated intercellular junctions of stratum corneum. The Ca^2+^ chelating activity of each molecule was confirmed through biochemical analysis, and its effect was verified at the molecular and skin tissue levels, and by an *in vivo* test. These results show the perspective of amino acids as effective molecules to help improve skin problems.

## Materials and methods

### Reagents

Amino acids, o-cresolphthalein, calcium chloride, and gluconolactone were purchased from Sigma-Aldrich. The porcine skin (Micropig, back skin, 2.5 × 2.5 cm^2^, 1 mm thickness) was obtained from MediKinetics (Pyeongtaek, South Korea). Phosphate buffered saline (PBS) was purchased from GIBCO (New York, USA). The human skin equivalent (Neoderm-E) was constructed by TEGO Science (Seoul, South Korea). The antibodies against Dsg1, Dsc1, and α-Tubulin were purchased from Thermo Fisher (MA, USA), Cloud-Clone (TX, USA), and Santa Cruz Biotechnology (MN, USA). The tape strip (D-Squame D100) for sampling stratum corneum was obtained from Cuderm (TX, USA). All other molecular biology reagents were purchased from commercial sources and were of analytical grade. The serine for human *in vivo* test was purchased from Evonik (Essen, Germany) and was of cosmetic grade. Human testing was in accordance with the Declaration of Helsinki.

### Analysis of Ca^2+^ chelating activity

The Ca^2+^ chelating activity of each amino acid was measured by a modified o-cresolphthalein complexone (CPC) method to evaluate concentrations of Ca^2+^ in solution [13]. As a stock solution, each amino acid was dissolved to its maximum concentration in distilled water and was serially diluted to several concentrations. The reaction mixtures (200 μL) contained Tris-HCl (150 mM, pH8.0), CaCl_2_ (0.5 mM), CPC (0.5 mM), and one of the amino acid solutions (16 μL of each stock). The absorbance of the reaction mixture was measured at 570 nm in a 96-well plate after 30 min incubation for equilibrium of molecular interaction. The concentration (IC_50_) of each amino acid for 50% inhibition of interaction of Ca^2+^ and CPC was estimated from triplicated experiments. EDTA was additionally analyzed as a positive chelating control.

### Analysis of *in vitro* exfoliating activity

To measure the exfoliating activity of each amino acid, pieces of porcine skin of 6 mm diameter were prepared and placed in each well of a 96-well plate. Solutions (1%) of amino acids were prepared in distilled water and the pH was adjusted to pH 6.0 using NaOH and HCl. After the solutions (200 μL) were added in each well with 0.1% Triton X-100, they were incubated at 37°C with 50% relative humidity for 16 h. Then, 10 μL of each well’s supernatant was placed in a hemocytometer (Paul Marienfeld GmbH & Co. KG) and number of cells released from the porcine skin was counted.

### Real time observation of exfoliation process

Lumps of stratum corneum were scraped off from the elbow skin of a volunteer using a surgical scalpel blade no.15 and suspended in distilled water (20 μL), followed by 2 h of mild stirring. After placing two cover glasses laterally on a slide glass, we put the solution including the lumps of stratum corneum on a gap between the two cover glasses and added an additional cover glass on the top. Then, 10% solution of serine (20 μL) was slowly added in the stratum corneum solution to provide a driving force for fluid flow so that separated pieces of stratum corneum could move away. The dissociation of stratum corneum by serine was observed under a microscope in real time.

### Observation of reversible binding of corneocytes

As the first step, individual corneocytes were obtained from stratum corneum of pig skin through overnight treatment of 10% serine solution. The corneocytes were centrifuged at 13000 rpm for 5 min and concentrated in a 1.5 mL microcentrifuge tube. After discarding the supernatant, corneocytes were dispersed by adding 1 mL water, and 100 μL was left for microscopic observation. In the remaining cells, calcium solution was added to final concentration of 5 mM, followed by incubation at 37 °C for 1 h. A portion of this sample was left for microscopic observation. The remaining solution was centrifuged and the supernatant was discarded. The corneocytes were repeatedly washed with distilled water to remove excess calcium. Finally, the corneocytes were again treated with 10% serine solution, incubated at 37 °C for 1 h and washed twice with distilled water. The corneocytes sampled at all steps were observed under a microscope.

### Immunoprecipitation

To investigate the status of Dsg1 and Dsc1 interaction, reconstructed human equivalent model (Neoderm-E) were treated with 10% serine or not topically for 3 days. Tissues were washed with cold PBS, and tissue pellets were incubated in RIPA buffer (50 mM Tris, pH 8.0, 150 mM NaCl, 1% NP-40, 1% Triton X-100) containing EDTA-free protease inhibitor cocktail (Sigma-Aldrich) for 15 min on ice. The extracts were separated by centrifugation at 13000 rpm for 30 min. The supernatants (1 mg) were used for immunoprecipitation using an anti-Dsg1 antibody for 12 h at 4 °C with shaking. Protein G sepharose 4B (Invitrogen) was then added and incubated for 4 h. Samples were washed four times with lysis buffer, boiled in 5× sample buffer containing β-mercaptoethanol, and subjected to electrophoresis on an 4–15% SDS-polyacrylamide gel (Bio-Rad). Separated proteins were transferred onto a nitrocellulose membrane and detected by anti-Dsc1 antibody and peroxidase-conjugated secondary antibody. Signals were detected using the Fusion FX 5 image system (Vilber Lourmat) and ECL western blotting detection reagents (Amersham). The experiment was performed three times. For morphological observations, a set of the equivalents was fixed in paraformaldehyde for 30 min and processed for conventional paraffin embedment. Sections were prepared in 5 μm thickness and were then stained with hematoxylin and eosin (H&E).

### Turnover determinations

Each ventral surface of forearm of ten healthy volunteers (25–50 years of age) was stained by 10% solution (350 μL) of dihydroxyacetone (DHA) using woven cotton gauzes (2 × 2 cm) and a Tegaderm Film (3 M, 8 × 12 cm^2^) for 8 h. After 2 days, the sites treated with DHA showed the development of visually darkest brownish coloration. Then, each site was topically applied with serine solutions twice a day for 10 days. Color recovery to the original skin color on each site was measured using a chromameter (CR-400, Konica Minolta). None of the volunteers showed any side effects such as erythema, itching, and burning sensation during and after the experiment.

### Statistical analysis

All values are expressed as means ± SD (standard deviation). Statistical differences were assessed by a two-tailed Student’s *t*-test between two groups. Probabilities less than 5% (*P < 0.05) or 1% (**P < 0.01) were considered to be statistically significant.

### Ethics statement

This study was conducted in accordance with the principles and guidelines expressed in the Declaration of Helsinki. The study design was approved by the institutional review board of LG Household and Health Care, Ltd. (IRB No. LGHH-20180601-AA-03, LGHH-20190221-AA-02) The institutional review board is operated independently including external evaluation members in accordance with the Korean Bioethics and Safety Act and certified by the Ministry of Health and Welfare of Korea. (Certification No. 1-20170421107-AB-N-01) All study participants provided informed written consent prior to study enrollment.

## Results and discussion

### Ca^2+^ chelating and exfoliating activity of amino acids

We measured the Ca^2+^ chelating activity of amino acids using a conventional o-cresolphthalein complexone (CPC) method. The binding of Ca^2+^ and amino acid was assessed by a decrease in the absorbance of Ca^2+^-CPC complex. (Table 1) Analysis of the relative potency indicated that amino acids with hydroxyl residue at the β-carbon position had relatively strong chelating power; these were serine and threonine as amino acids, and carnitine as a derivative of amino acid. The molecular structure of these molecules has similar functional moiety for Ca^2+^ chelation as that of β-hydroxy acids such as salicylic acid, malic acid, and citric acid. Cysteine, histidine, and aspartate and glutamate that have sulfhydryl, imidazole, or di-carboxyl acid moieties, respectively, were also confirmed to have Ca^2+^ chelating activity. In physiological system, these molecules have been reported to play a role as metal-coordinating modulators inside proteins [14–17]. The other amino acids did not inhibit the Ca^2+^-CPC complex even at the maximum dissolution concentration. (S1 Table).

**Table 1:** Amino acids with Ca^2+^ chelating activity.

Name	Functional Group	IC_50_ (μM)
**Gly**	COOH, NH_2_	680 ± 1.5
**Ser**	COOH, OH, NH_2_	190 ± 1.8
**Thr**	COOH, OH, NH_2_	180 ± 1.0
**Cys**	COOH, SH, NH_2_	210 ± 0.1
**Asp**	2 COOH, NH_2_	740 ± 0.7
**Glu**	2 COOH, NH_2_	2900 ± 170
**His**	COOH, NH_2_, Imidazole	760 ± 16
Car*	COOH, OH, NH_2_	160 ± 0.3
**EDTA**^#^	4 COOH	2.5 ± 0.01

Functional groups of each molecule are presented. IC_50_ of amino acids for the interaction of Ca^2+^ and CPC was measured in pH 8.0.

*Carnitine as a derivative of lysine was additionally tested based on its molecular property for Ca^2+^ chelation.

^#^EDTA was analyzed as a positive control.

The Ca^2+^ chelating activity of amino acids was about two orders lower than that of EDTA.

Each value represents mean ± SD in triplicated experiments.

**Fig 1:**
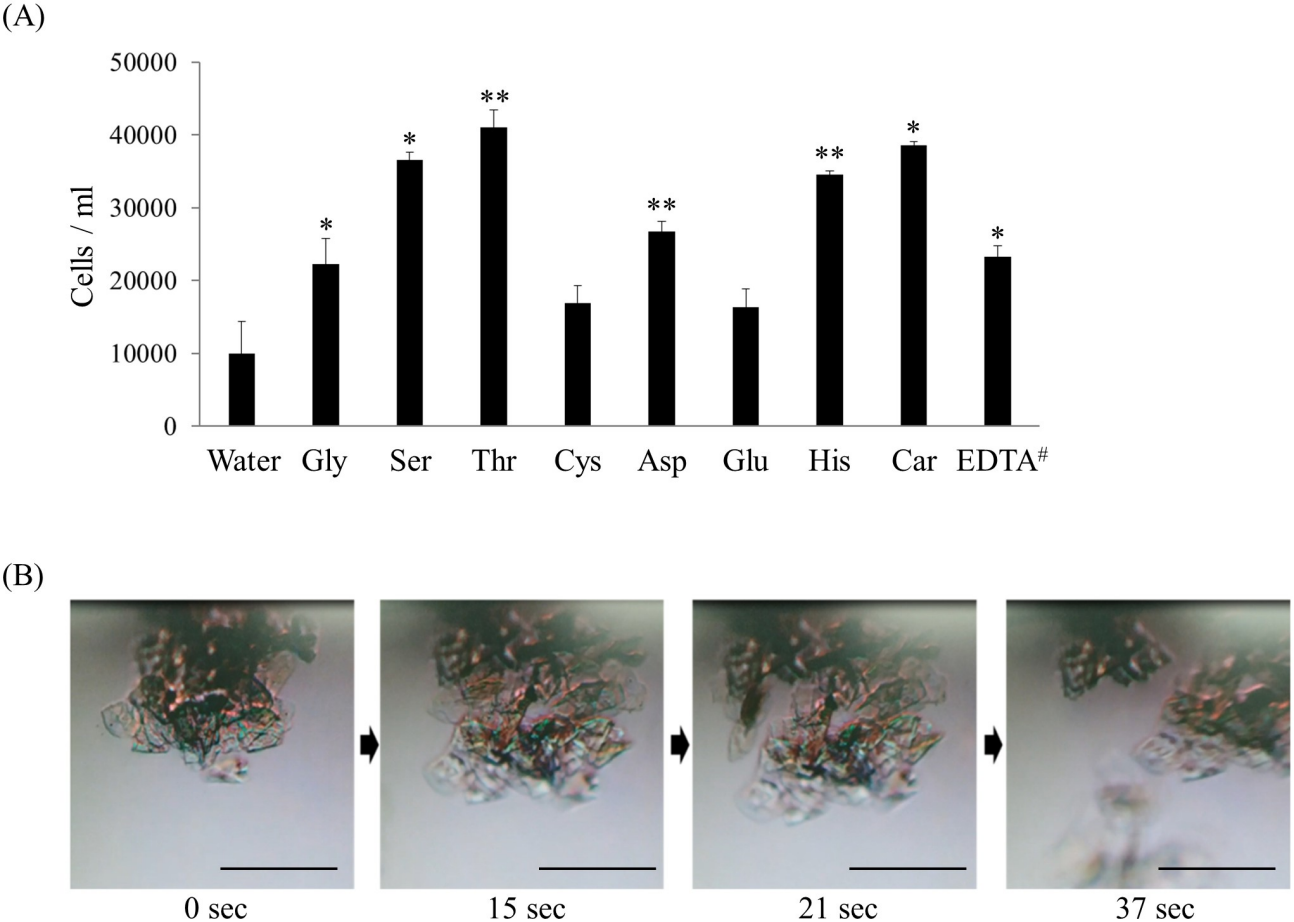
Exfoliating activity of Ca^2+^ chelating amino acids. (A) Number of cells chemically released from porcine skin was compared with that of distilled water as a negative control. Car means carnitine. All molecules were tested at 1% except ^#^EDTA was tested at 10%. Each value represents mean ± SD, *P<0.05 and **P < 0.01 vs. the water control value in triplicated experiments. (B) Real time dissociation of stratum corneum was observed in the presence of serine. Scale bar 100 μm.

Exfoliating activity of each amino acid was evaluated by counting the number of cells that were chemically released from porcine skin after treatment with amino acids, as a mimicked method of actual exfoliation process. (Fig 1A and S1 Fig) These observations also suggest that amino acids with strong Ca^2+^ chelating power tend to exert high exfoliating activity. To observe the exfoliation process in real time, we scraped stratum corneum from the elbow skin of a human volunteer. After being fully hydrated in water, the samples were incubated with serine as a representative of an amino acid with high Ca^2+^ chelating activity and exfoliating activity. Within one minute of addition of serine, the stratum corneum was separated into pieces, as seen under the microscope. (Fig 1B) Taken together, our observations reveal that amino acids can promote the exfoliation of stratum corneum. In the case of EDTA, its exfoliating activity was lower than that of amino acids such as serine, even though it was a powerful chelator. And the cellular dissociation of stratum corneum by EDTA was also not observed within the measurement time (S2 Fig). EDTA has strong negative charge at neutral pH and its molecular weight is relatively large compared to amino acids. This feature appears to cause poor interaction with negatively charged stratum corneum [18, 19], thereby resulting in low exfoliating activity.

### Disruption of Ca^2+^-mediated binding of stratum corneum by amino acid

**Fig 2:**
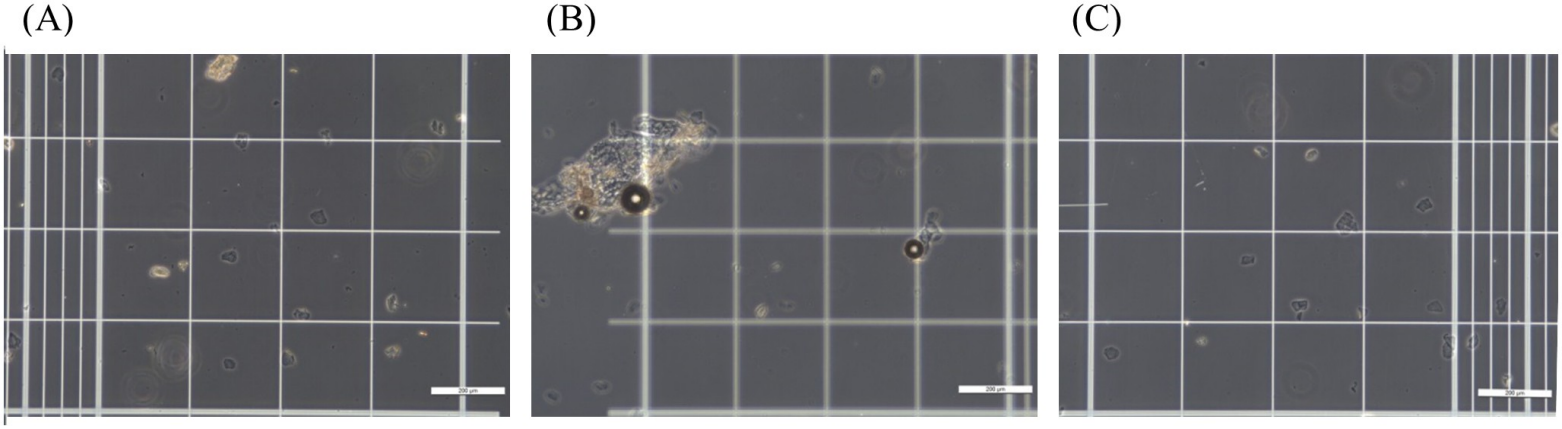
Ca^2+^-mediated reversible binding of corneocytes. (A) Corneocytes exists in dispersed state. (B) After Ca^2+^ addition, randomly bound aggregates of corneocytes are observed. (C) After serine treatment, corneocytes are separated again. Scale bar 200 μm. All data were measured on a hemocytometer (250 × 250 μm^2^).

Previous studies reported that cells expressing cadherins showed robust cell aggregation under high Ca^2+^ and reversible separation in presence of Ca^2+^ chelators [2,9]. This phenomenon was also evaluated under conditions of Ca^2+^ and amino acid. Individual corneocytes from stratum corneum were obtained through overnight incubation with serine. After washing the cells with water, they were treated with CaCl_2_ solution, which resulted in aggregation of the cells (Fig 2B). Further, additional treatment with serine resulted in separation of the aggregates (Fig 2C). These results indicate that adhesion of stratum corneum is modulated in a Ca^2+^-dependent manner and Ca^2+^ chelating amino acids can disrupt this adhesion.

**Fig 3:**
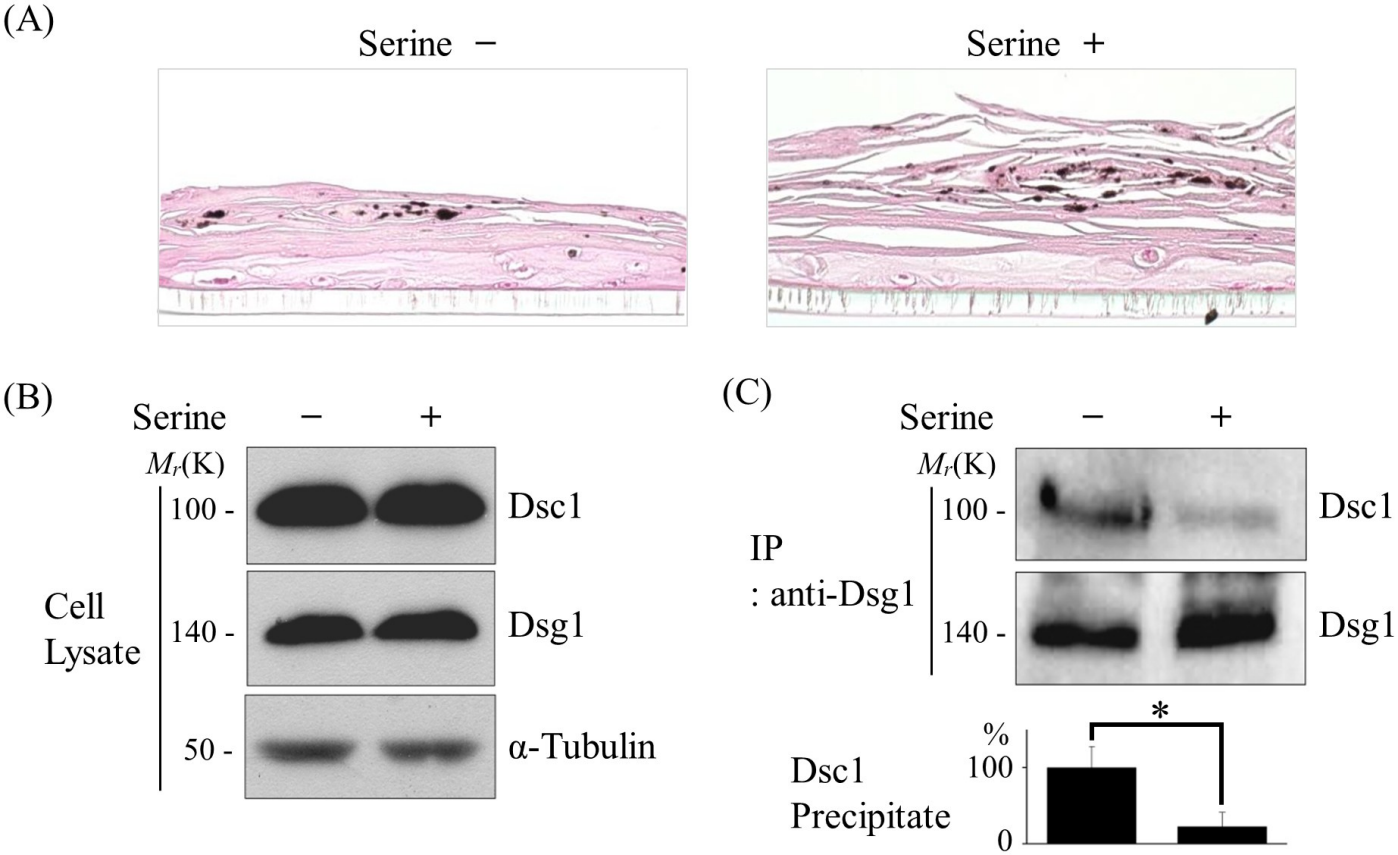
The coimmunoprecipitation of Dsg and Dsc. (A) Sections of skin equivalents were stained with hematoxylin and eosin (H&E) (B) Expression level of Dsg1 and Dsc1 was unaffected by the presence or absence of serine. Expression level of α-Tubulin was verified as a control for loading of same amount of the cell lysates in gel electrophoresis. (C) Immunoprecipitates by anti-Dsg1 antibody were analyzed by immunoblotting with anti-Dsg1 and anti-Dsc1. In the presence of serine, the amount of coimmunoprecipitated Dsc1 decreased. Bars indicate means ± SD of relative densitometric values of Dsc1 from the repeated experiments (n = 3). *P < 0.05 vs. the untreated control.

To further investigate the influence of amino acids at a molecular level, we tested whether amino acids interfere with the interaction of cadherins. The stratum corneum of an artificial skin equivalent was lysed in the presence of serine. Fig 3A shows that layers of stratum corneum partially departed from the skin equivalent after serine treatment. The lysates of the serine-treated equivalent were immunoprecipitated with anti-Dsg1 antibody and the presence of Dsc1 in the immunoprecipitants was detected by western blot analysis using anti-Dsc1 antibody. The data revealed that treatment of serine did not alter the expression levels of Dsg1 and Dsc1 (Fig 3B), and instead reduced the amount of Dsg/Dsc complexes, implying detachment of the two cadherins (Fig 3C). These observations show that amino acid (serine) chelates Ca^2+^ in stratum corneum, dissociating the intercellular junctions, and thereby breaking down the tissue into fragments. The same results were obtained in several repeated experiments (S3 Fig).

### Acceleration of stratum corneum turnover by amino acid

**Fig 4:**
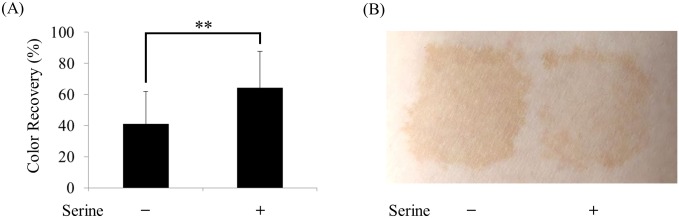
Color recovery of DHA-stained stratum corneum by amino acid. (A) Color recovery of 5% serine-treated site and untreated site after 10 days is presented. Data represent the means ± SD and **P < 0.01 between the two groups. (B) A representative image of de-pigmented site of a volunteer after 10 days is shown.

To confirm exfoliating activity of amino acid on real human skin, we investigated the elimination rate of stratum corneum pigmentation induced by dihydroxyacetone (DHA), similarly to previously reported methods [20–22]. After pigmentation on the ventral surface of each forearm of volunteers, serine was topically applied to the pigmented site and decoloration of the site was measured for 10 days. As shown in Fig 4, the serine-treated site recovered faster than the untreated site, suggesting that amino acid with Ca^2+^ chelating activity can exert an effect on real human skin, accelerating the turnover of stratum corneum. The DHA staining was not observed to be chemically discolored by serine (S4 Fig).

Amino acids are very common in the stratum corneum, and most of them are produced by extensive proteolysis of proteins such as filaggrin [23]. In particular, the concentration of serine is reported to be about 1–2% by weight of the stratum corneum [24, 25]. Our additional *in vivo* experiment showed that serine had significant turnover-accelerating activity at concentrations above 0.5% (S5 Fig). This seems to be a reasonable level of concentration that can lead to physiological changes in the stratum corneum.

## Conclusions

In this study, we demonstrated that amino acids, such as serine, disrupt Ca^2+^-dependent adhesion of stratum corneum. Our observations indicate that this function could be attributed to the Ca^2+^ chelating property of amino acids. Molecules with similar Ca^2+^ ion chelating abilities are expected to have similar efficacy in stratum corneum. Further studies are necessary to examine the effects of such molecules, including amino acids, on human skin.

## Supporting information

S1 Table Ca^2+^ chelating activity of amino acids. IC_50_ of amino acids for the interaction of Ca^2+^ and CPC was measured in pH 8.0. *Car means carnitine. ^#^EDTA was additionally tested as a positive control. Each value represents mean ± SD in triplicated experiments.
(PDF)

S1 Fig Exfoliating activity of amino acids. Number of cells chemically released from porcine skin was compared with that of distilled water as a negative control. Carnitine (Car) as a derivative of lysine was additionally tested. EDTA was tested at 5% and 10% concentrations and showed unexpectedly weak exfoliating activity. Each value represents mean ± SD, *P<0.05 and **P < 0.01 vs. the water control value in triplicated experiments.
(TIF)

S2 Fig Real time observation of stratum corneum. Lumps of stratum corneum was placed in the presence of distilled water (A) and 10% EDTA solution (B), respectively. Unlike the serine, the separation of the stratum corneum was not observed within the measurement time. Scale bar 100 μm.
(TIF)

S3 Fig The coimmunoprecipitation of Dsg and Dsc. Two independent experiments were performed in the same way. (A, C) Western blotting of cell lysates showed that serine did not affect the expression levels of Dsg1 and Dsc1. (B, D) Immunoprecipitation assay using anti-Dsg1 antibody showed that the amount of coimmunoprecipitated Dsc1 decreased in the presence of serine. The same results were seen in repeated experiments. The entire blots are displayed.
(TIF)

S4 Fig Serine does not chemically decolorize the DHA product. In order to confirm that the color recovery of DHA-stained stratum corneum by serine is not due to chemical decolorization, tape strips sampling the pigmented site were treated with serine. There was no change in the color of DHA staining even after 2 days. This result shows that decolorization of the pigment resulted from acceleration of stratum corneum turnover by serine. Each value represents the means ± SD of lightness (L) measured using a chromameter on white background and *P<0.05 vs. the tape that sampled unpigmented site. Experiments were carried out in triplicate, and an image of each is shown.
(TIF)

S5 Fig Color recovery of DHA-stained stratum corneum by serine. Color recovery of 0.1–5% serine-treated and untreated sites after 10 days is presented. Significant efficacy was observed at concentrations above 0.5%. Data represent the means ± SD and *P<0.05 and **P < 0.01 vs. the untreated site.
(TIF)

S1 Dataset Underlying data used to reach the conclusions drawn in the manuscript. (PDF)
