# Supplementary material for: Amino acids disrupt calcium-dependent adhesion of stratum corneum
Source: PLoS One. 2019 Apr 16;14(4):e0215244. doi: 10.1371/journal.pone.0215244 (PMC6467405; doi:10.1371/journal.pone.0215244)
Supplement: S1 Table — IC50 of amino acids for the interaction of Ca2+ and CPC was measured in pH 8.0. *Car means carnitine. #EDTA was additionally tested as a positive control. Each value represents mean ± SD in triplicated experiments. (PDF) [file pone.0215244.s001.pdf]

Name	Functional Group	IC_{50} (μM)
**Gly**	COOH, NH_2	680 ± 1.5
**Ala**	COOH, NH_2	> 150000
**Ser**	COOH, OH, NH_2	190 ± 1.8
**Thr**	COOH, OH, NH_2	180 ± 1.0
**Cys**	COOH, SH, NH_2	210 ± 0.1
**Val**	COOH, NH_2	> 40000
**Leu**	COOH, NH_2	> 13000
**Ile**	COOH, NH_2	> 21000
**Met**	COOH, NH_2, Thioether	> 30000
**Pro**	COOH, NH_2	> 110000
**Phe**	COOH, NH_2	> 13000
**Tyr**	COOH, OH, NH_2	> 210
**Trp**	COOH, NH_2	> 5300
**Asp**	2 COOH, NH_2	740 ± 0.7
**Glu**	2 COOH, NH_2	2900 ± 170
**Asn**	COOH, CO, 2 NH_2	> 18000
**Gln**	COOH, CO, 2 NH_2	> 23000
**His**	COOH, NH_2, Imidazole	760 ± 16
**Lys**	COOH, 2 NH_2	> 320000
**Arg**	COOH, NH_2, Guanidino	> 84000
**Car***	COOH, OH, NH_2	160 ± 0.3
**EDTA#**	4 COOH	2.5 ± 0.01
