# Supplementary figures and images for: Amino acids disrupt calcium-dependent adhesion of stratum corneum
Source: PLoS One. 2019 Apr 16;14(4):e0215244. doi: 10.1371/journal.pone.0215244 (PMC6467405; doi:10.1371/journal.pone.0215244)

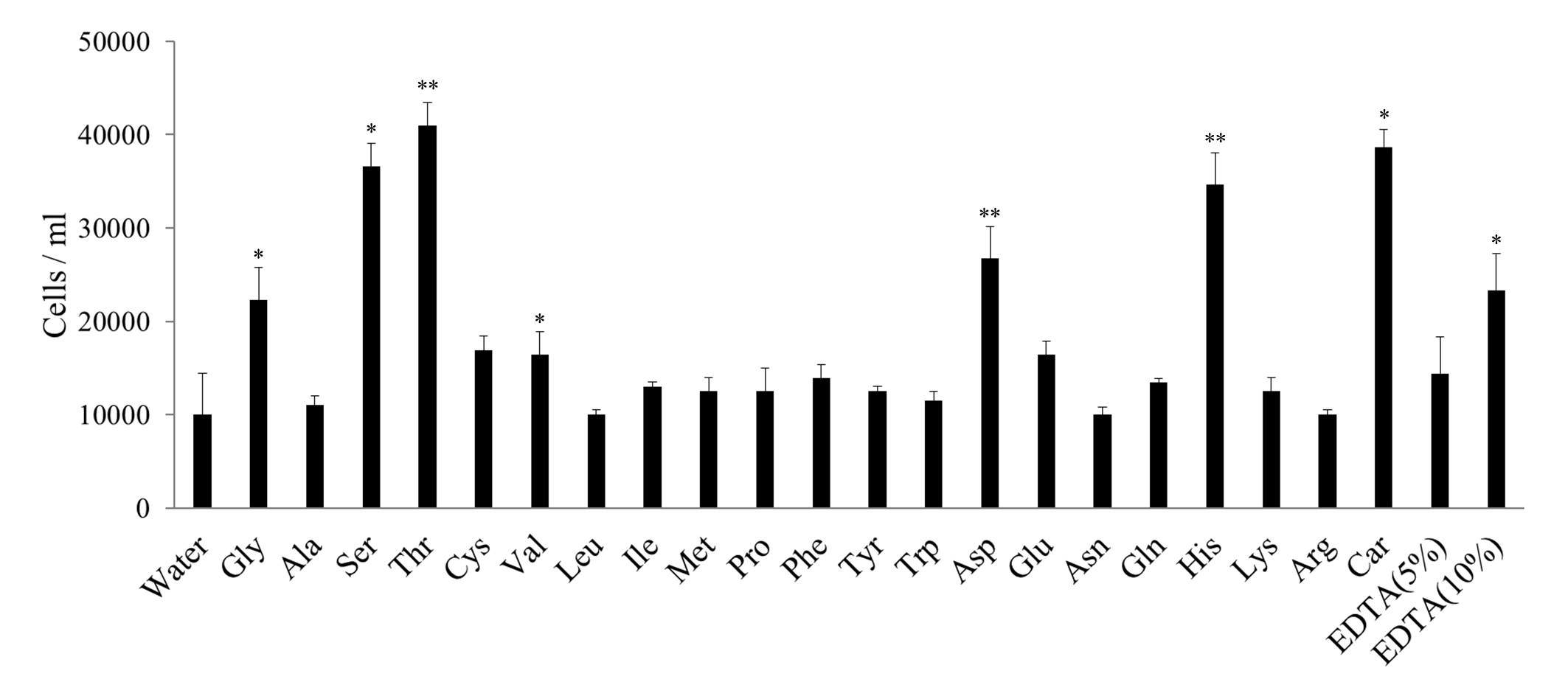

Supplement: S1 Fig — Number of cells chemically released from porcine skin was compared with that of distilled water as a negative control. Carnitine (Car) as a derivative of lysine was additionally tested. EDTA was tested at 5% and 10% concentrations and showed unexpectedly weak exfoliating activity. Each value represents mean ± SD, *P<0.05 and **P < 0.01 vs. the water control value in triplicated experiments. (TIF) [file pone.0215244.s002.tif]

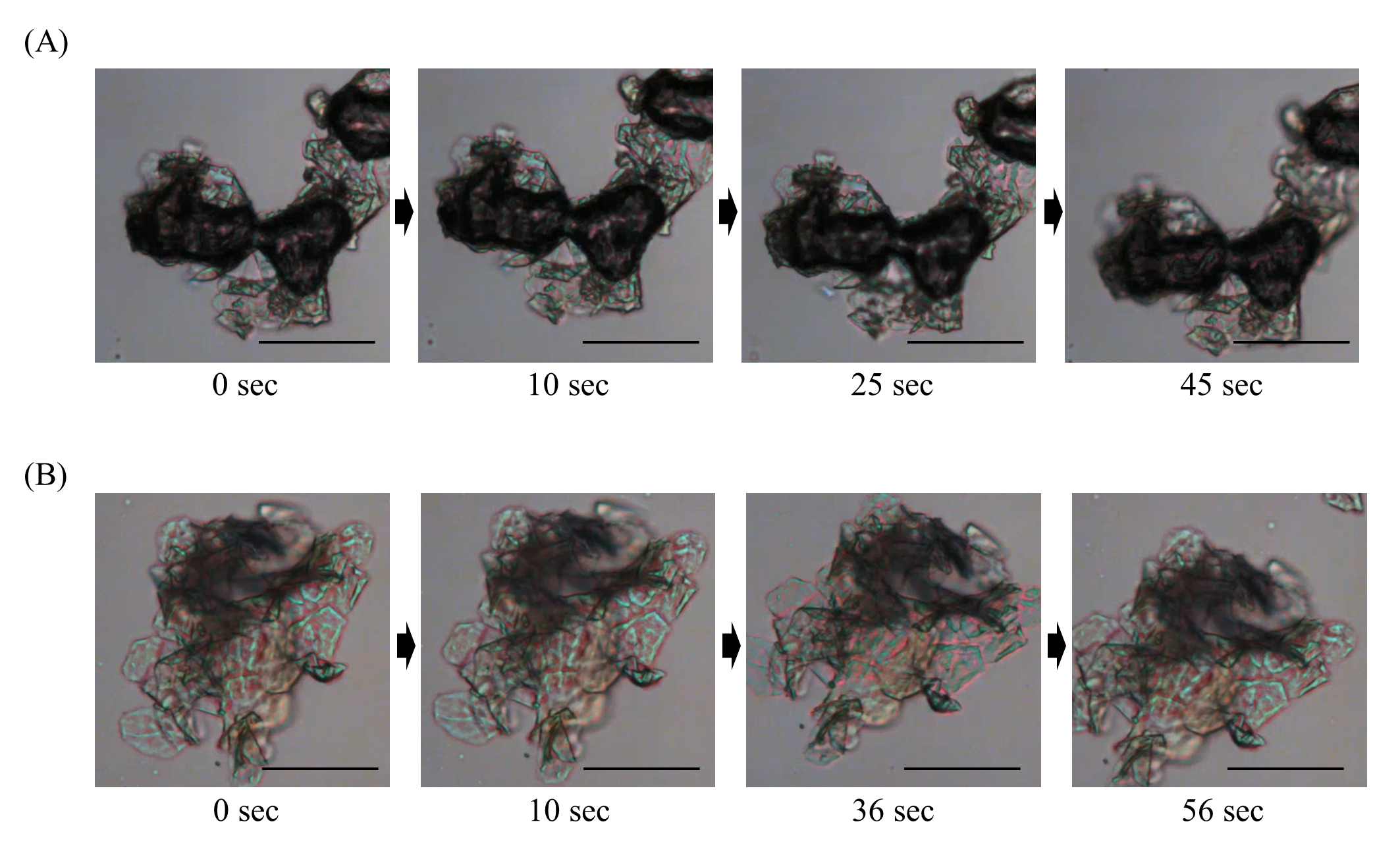

Supplement: S2 Fig — Lumps of stratum corneum was placed in the presence of distilled water (A) and 10% EDTA solution (B), respectively. Unlike the serine, the separation of the stratum corneum was not observed within the measurement time. Scale bar 100 μm. (TIF) [file pone.0215244.s003.tif]

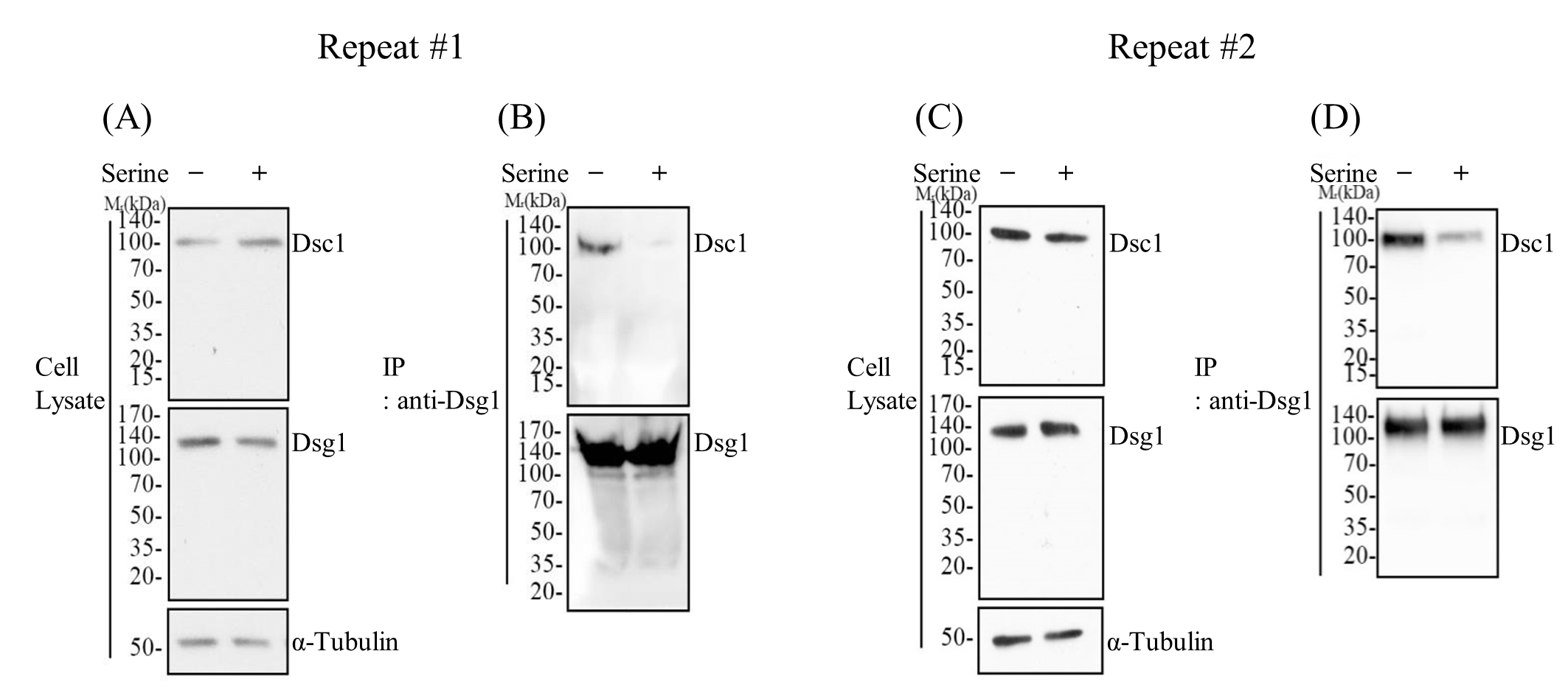

Supplement: S3 Fig — Two independent experiments were performed in the same way. (A, C) Western blotting of cell lysates showed that serine did not affect the expression levels of Dsg1 and Dsc1. (B, D) Immunoprecipitation assay using anti-Dsg1 antibody showed that the amount of coimmunoprecipitated Dsc1 decreased in the presence of serine. The same results were seen in repeated experiments. The entire blots are displayed. (TIF) [file pone.0215244.s004.tif]

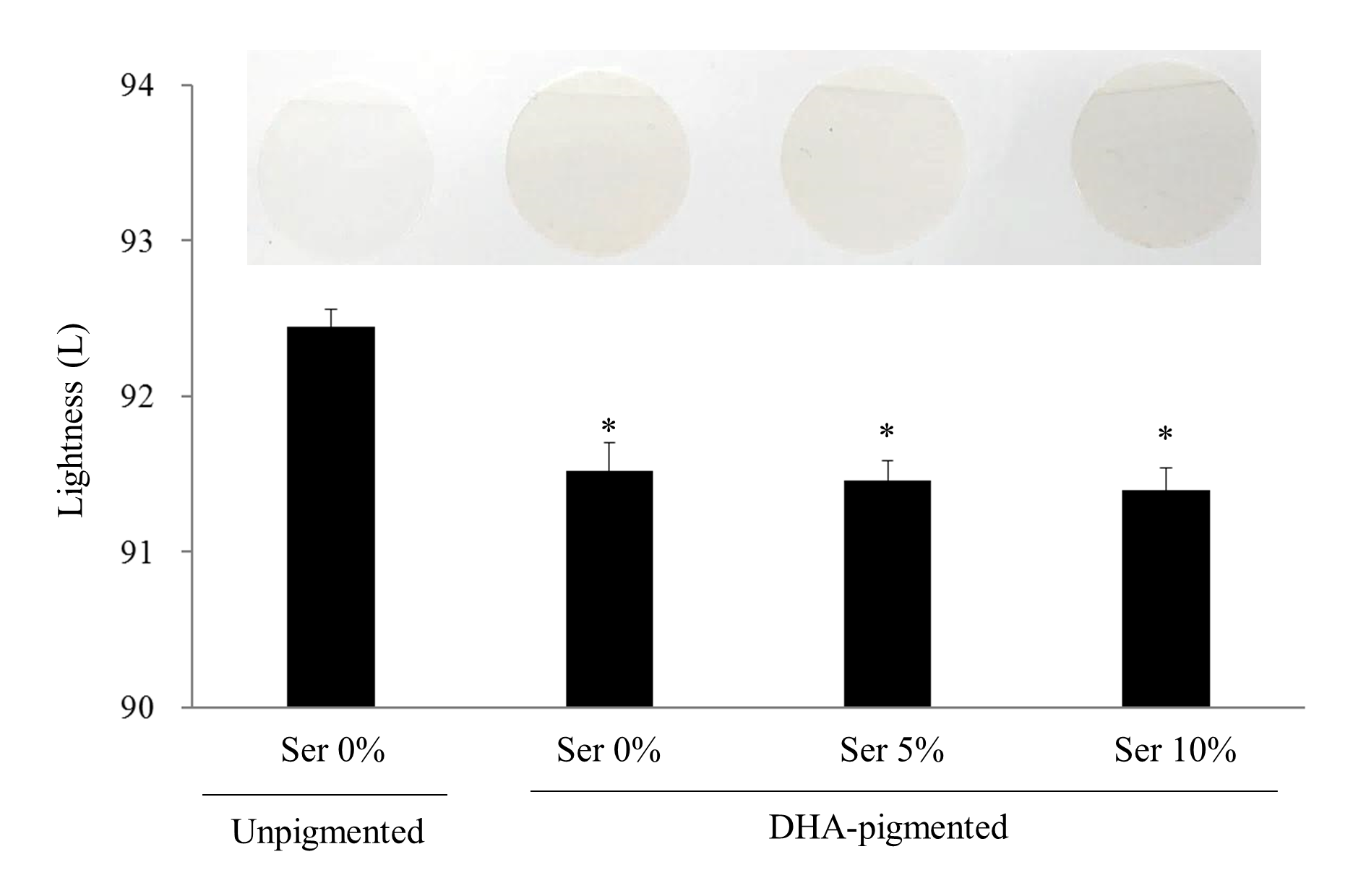

Supplement: S4 Fig — In order to confirm that the color recovery of DHA-stained stratum corneum by serine is not due to chemical decolorization, tape strips sampling the pigmented site were treated with serine. There was no change in the color of DHA staining even after 2 days. This result shows that decolorization of the pigment resulted from acceleration of stratum corneum turnover by serine. Each value represents the means ± SD of lightness (L) measured using a chromameter on white background and *P<0.05 vs. the tape that sampled unpigmented site. Experiments were carried out in triplicate, and an image of each is shown. (TIF) [file pone.0215244.s005.tif]

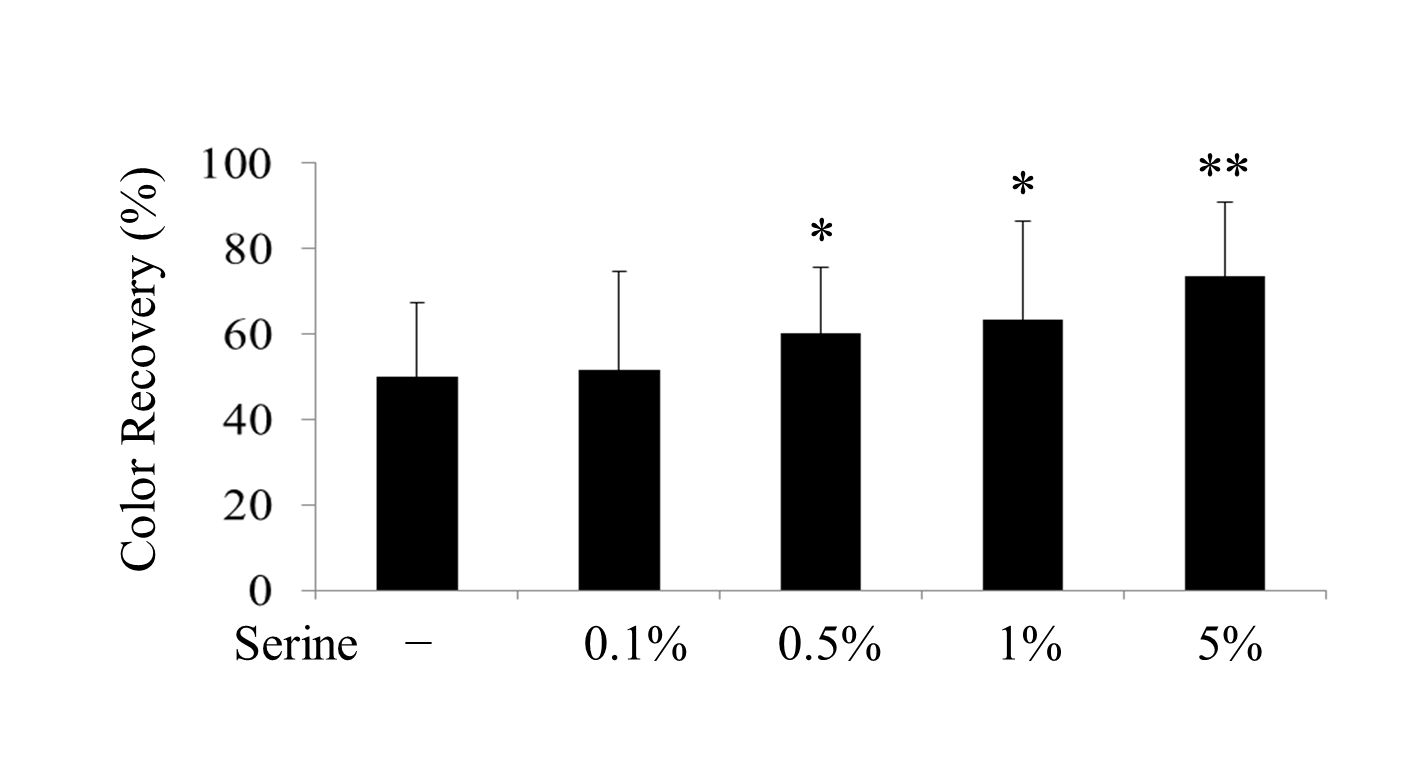

Supplement: S5 Fig — Color recovery of 0.1–5% serine-treated and untreated sites after 10 days is presented. Significant efficacy was observed at concentrations above 0.5%. Data represent the means ± SD and *P<0.05 and **P < 0.01 vs. the untreated site. (TIF) [file pone.0215244.s006.tif]
