# Supplementary material for: Amino acids disrupt calcium-dependent adhesion of stratum corneum
Source: PLoS One. 2019 Apr 16;14(4):e0215244. doi: 10.1371/journal.pone.0215244 (PMC6467405; doi:10.1371/journal.pone.0215244)
Supplement: S1 Dataset — (PDF) [file pone.0215244.s007.pdf]

1) Data set for Table1 and S1 table. #Each value means the relative % compared with absorbance value of $CaCl_2$ (0.5 mM) control.

Gly (μM)	1332	999	666	333	133	IC_{50} (μM) of Gly		
Trial#1 (%)	30.9	39.6	53.1	69.5	85.8	681	Mean 682	SD 1.48
Trial#2 (%)	31.0	39.6	53.3	69.5	86.0	680		
Trial#3 (%)	31.0	39.8	53.3	69.7	86.1	683		

Ser (μM)	952	714	476	238	95.2	IC_{50} (μM) of Ser		
Trial#1 (%)	4.16	15.4	23.8	36.7	67.9	195	Mean 194	SD 1.75
Trial#2 (%)	4.16	15.4	24.4	36.2	67.8	195		
Trial#3 (%)	4.16	15.5	22.6	36.5	68.0	192		

Thr (μM)	420	315	210	105	42.0	IC_{50} (μM) of Thr		
Trial#1 (%)	25.4	30.8	48.2	64.6	84.1	182	Mean 183	SD 1.01
Trial#2 (%)	25.5	30.9	48.3	64.9	84.4	183		
Trial#3 (%)	25.5	30.9	48.5	65.0	84.4	184		

Cys (μM)	330	248	165	82.5	33.0	IC_{50} (μM) of Cys		
Trial#1 (%)	36.1	42.2	58.2	77.6	86.1	209	Mean 209	SD 0.13
Trial#2 (%)	36.2	42.3	58.3	77.7	86.2	209		
Trial#3 (%)	36.2	42.1	58.2	77.5	86.1	210		

Asp (μM)	349	174	34.9	IC_{50} (μM) of Asp		
Trial#1 (%)	74.9	87.3	94.9	741	Mean 741	SD 0.69
Trial#2 (%)	74.8	88.3	95.1	740		
Trial#3 (%)	74.9	88.2	95.2	741		

Glu (μM)	508	381	254	127	50.8	IC_{50} (μM) of Glu		
Trial#1 (%)	89.9	93.4	93.6	97.2	97.6	2950	Mean 2911	SD 174.8
Trial#2 (%)	90.6	93.2	94.1	97.1	98.0	3062		
Trial#3 (%)	89.0	93.7	94.1	97.0	97.8	2719		

His (μM)	121	80.6	40.3	16.1	IC_{50} (μM) of His		
Trial#1 (%)	87.4	89.4	92.7	93.0	763	Mean 756	SD 16.0
Trial#2 (%)	87.5	89.6	92.5	93.3	767		
Trial#3 (%)	87.3	89.6	92.8	93.2	738		

Car (μM)	620	465	310	155	62.0	IC_{50} (μM) of Car		
Trial#1 (%)	2.80	13.0	29.1	53.5	78.0	158	Mean 159	SD 0.29
Trial#2 (%)	3.10	13.1	28.5	54.4	78.8	159		
Trial#3 (%)	3.20	14.1	28.3	54.0	79.0	159		

EDTA (μM)	2.43	1.82	1.21	0.61	IC_{50} (μM) of EDTA		
Trial#1 (%)	51.4	70.1	87.7	99.6	2.53	Mean 2.54	SD 0.01
Trial#2 (%)	52.1	66.8	82.8	93.4	2.55		
Trial#3 (%)	51.9	67.1	81.3	92.5	2.55		

2) Data set for Fig 1 and S1 Fig. Number of cells chemically released from porcine skin after treatment of each molecule.

	Cells / 10 μL					Cells / 1 mL		t-test
Molecules	#1	#2	#3	Mean	SD	Mean	SD	
Water	104	53	141	99	44.2	9900	4420	-
Gly	188	224	257	223	34.5	22300	3450	0.040
Ala	105	103	121	110	9.87	11000	987	0.668
Ser	370	389	340	366	24.7	36600	2470	0.021
Thr	433	384	413	410	24.6	41000	2464	0.004
Cys	154	170	184	169	15.0	16900	1500	0.097
Val	175	136	182	164	24.8	16400	2480	0.035
Leu	100	106	96	101	5.03	10100	503	0.967
Ile	132	124	133	130	4.93	13000	493	0.315
Met	130	108	136	125	14.7	12500	1470	0.281
Pro	98	131	146	125	24.6	12500	2460	0.433
Phe	144	122	151	139	15.1	13900	1510	0.145
Tyr	119	129	126	125	5.13	12500	513	0.444
Trp	109	126	110	115	9.54	11500	954	0.659
Asp	268	232	301	267	34.5	26700	3450	0.001
Glu	163	150	179	164	14.5	16400	1450	0.064
Asn	101	107	92	100	7.55	10000	755	0.984
Gln	131	140	133	135	4.73	13500	473	0.331
His	381	311	345	346	35.0	34600	3500	0.008
Lys	110	141	125	125	15.5	12500	1550	0.498
Arg	103	98	94	98	4.51	9800	451	0.973
Car	402	392	364	386	19.7	38600	1970	0.014
EDTA(5%)	99	164	170	144	39.4	14400	3940	0.321
EDTA(10%)	261	187	250	233	39.9	23300	3990	0.011

3) Data set for Fig 3C. Relative densitometric values of Dsc1 from the repeated experiments.

Difference in signal intensity between background and DSC1 band was displayed.

	Serine -	Serine +
Trial#1 (a.u.*)	54.2	1.79
Trial#2 (a.u.*)	95.1	27.7
Trial#3 (a.u.*)	70.5	20.3
Mean (a.u.*)	73.3	16.6
Relative mean (%)	100	22.7
Relative SD (%)	28.2	18.2
t-test	-	0.016

* The a.u. means arbitrary unit.

4) Data set for Fig 4. Recovery (%) of the L value measured using a chromameter

after treatment for 10 days.

Volunteers	Serine -	Serine +
#1	21.0	44.9
#2	29.2	100
#3	17.3	43.9
#4	37.1	70.2
#5	69.6	100
#6	18.9	37.2
#7	37.9	49.6
#8	79.9	92.4
#9	45.0	55.3
#10	56.8	50.5
Mean	41.2	64.4
SD	20.5	23.2
t-test	-	0.0058

5) Data set for S4 Fig. The L values of tape strips measured using a chromameter.

	Unpigmented	DHA-pigmented		
	Serine 0%	Serine 0%	Serine 5%	Serine 10%
Trial#1	92.5	91.3	91.3	91.2
Trial#2	92.3	91.7	91.6	91.6
Trial#3	92.6	91.6	91.5	91.4
Mean	92.4	91.5	91.5	91.4
SD	0.115	0.185	0.129	0.142
t-test	-	0.036	0.021	0.025

6) Data set for S5 Fig. Recovery (%) of the L value measured using a chromameter after treatment for 10 days.

Volunteers	Serine -	Serine 0.1%	Serine 0.5%	Serine 1%	Serine 5%
#1	55.8	65.1	63.2	69.8	83.4
#2	19.0	13.7	26.6	12.1	51.7
#3	67.3	65.2	71.5	70.5	75.1
#4	38.1	32.0	55.3	85.3	99.7
#5	70.5	82.1	90.0	98.3	100
#6	45.7	60.2	66.6	64.5	77.1
#7	23.5	13.6	49.0	35.9	52.1
#8	68.0	72.8	60.2	70.5	75.8
#9	53.5	43.6	59.1	57.1	50.6
#10	58.6	67.1	60.9	68.5	70.0
Mean	50.0	51.6	60.2	63.3	73.5
SD	17.3	23.2	15.3	23.0	17.3
t-test	-	0.605	0.011	0.023	0.0028
